# Supplementary material for: Nanoscale structural organization and stoichiometry of the budding yeast kinetochore
Source: J Cell Biol. 2023 Jan 27;222(4):e202209094. doi: 10.1083/jcb.202209094 (PMC9929930; doi:10.1083/jcb.202209094)
Supplement: Table S1 — shows the comparison of the available distance measurements from this article and Joglekar et al. (2009). [file JCB_202209094_TableS1.docx]

**Table S1. Comparison of the available distance measurements from this article and Joglekar et al. 2009.** Due to differences in reference points all distances were unified to the distance from Ndc80 C-terminus for clarity.

| **Protein pair (with Ndc80)** | **Distances (nm)** | |
| --- | --- | --- |
|  | **This work** | **Joglekar et al. 2009** |
| Ask1 | 30.7 | 27 |
| Spc105 | -13.6 | -16 |
| Nsl1 | -7.1 | -22 |
| Nnf1 | -8.8 | -14 |
| Mtw1 | -9.3 | -24 |
| Dsn1 | -10.5 | -24 |
| Ctf19 | -28.5 | -33 |
| Okp1 | -27.0 | -30 |
