# Supplementary material for: Nanoscale structural organization and stoichiometry of the budding yeast kinetochore
Source: J Cell Biol. 2023 Jan 27;222(4):e202209094. doi: 10.1083/jcb.202209094 (PMC9929930; doi:10.1083/jcb.202209094)
Supplement: Table S2 — shows the yeast strains created and used in this study. [file JCB_202209094_TableS2.docx]

**Table S2. The yeast strains created and used in this study.** All are based on the MKY100 strain (S288c derivative; Kaksonen Lab) with the following genetic background: MATa, ura3-52, his3𝛥200, leu3-52, lys2-801.

| **Strain name** | **Genotype** |
| --- | --- |
| Spc42-GFP/Ndc80-SNAP/Ask1-mMaple | SPC42-GFP::kanMX4, SPC105-SNAP::hphNT1,ASK1-mMaple::HIS3MX6 |
| Spc42-GFP/Spc105-SNAP/Cep3-mMaple | SPC42-GFP::kanMX4, SPC105-SNAP::hphNT1,CEP3-mMaple::HIS3MX6 |
| Spc42-GFP/Spc105-SNAP/Cse4-mMaple | SPC42-GFP::kanMX4, SPC105-SNAP::hphNT1,CSE4-mMaple::HIS3MX6 |
| Spc42-GFP/Spc105-SNAP/Cnn1-mMaple | SPC42-GFP::kanMX4, SPC105-SNAP::hphNT1,CNN1-mMaple::HIS3MX6 |
| Spc42-GFP/Spc105-SNAP/Chl4-mMaple | SPC42-GFP::kanMX4, SPC105-SNAP::hphNT1,CHL4-mMaple::HIS3MX6 |
| Spc42-GFP/Spc105-SNAP/Ctf19-mMaple | SPC42-GFP::kanMX4, SPC105-SNAP::hphNT1,CTF19-mMaple::HIS3MX6 |
| Spc42-GFP/Spc105-SNAP/Dsn1-mMaple | SPC42-GFP::kanMX4, SPC105-SNAP::hphNT1,DSN1-mMaple::HIS3MX6 |
| Spc42-GFP/Spc105-SNAP/Mif2-mMaple | SPC42-GFP::kanMX4, SPC105-SNAP::hphNT1,MIF2-mMaple::HIS3MX6 |
| Spc42-GFP/Spc105-SNAP/Mtw1-mMaple | SPC42-GFP::kanMX4, SPC105-SNAP::hphNT1,MTW1-mMaple::HIS3MX6 |
| Spc42-GFP/Spc105-SNAP/Ndc80-mMaple | SPC42-GFP::kanMX4, SPC105-SNAP::hphNT1,NDC80-mMaple::HIS3MX6 |
| Spc42-GFP/Spc105-SNAP/Nnf1-mMaple | SPC42-GFP::kanMX4, SPC105-SNAP::hphNT1,NNF1-mMaple::HIS3MX6 |
| Spc42-GFP/Spc105-SNAP/Nsl1-mMaple | SPC42-GFP::kanMX4, SPC105-SNAP::hphNT1,NSL1-mMaple::HIS3MX6 |
| Spc42-GFP/Spc105-SNAP/Spc25-mMaple | SPC42-GFP::kanMX4, SPC105-SNAP::hphNT1,SPC25-mMaple::HIS3MX6 |
| Spc42-GFP/Ndc80-SNAP/Ctf19-mMaple | SPC42-GFP::kanMX4, SPC105-SNAP::hphNT1,CTF19-mMaple::HIS3MX6 |
| Ndc80-SNAP/Nuf2-mMaple | NDC80-SNAP::hphNT1, NUF2-mMaple::HIS3MX6 |
| Ndc80-GFP/Cep3-mMaple | NDC80-GFP::kanMX4, CEP3-mMaple::HIS3MX6 |
| Ndc80-GFP/Cse4-mMaple | NDC80-GFP::kanMX4, CSE4-mMaple::HIS3MX6 |
| Ndc80-GFP/Cse4-mMaple-Cse4 | NDC80-GFP::kanMX4, cse4::CSE4-mMaple-CSE4::HIS3MX6 |
| Ndc80-GFP/Cnn1-mMaple | NDC80-GFP::kanMX4, CNN1-mMaple::HIS3MX6 |
| Ndc80-GFP/Chl4-mMaple | NDC80-GFP::kanMX4, CHL4-mMaple::HIS3MX6 |
| Ndc80-GFP/Ctf19-mMaple | NDC80-GFP::kanMX4, CTF19-mMaple::HIS3MX6 |
| Ndc80-GFP/Dsn1-mMaple | NDC80-GFP::kanMX4, DSN1-mMaple::HIS3MX6 |
| Ndc80-GFP/Mif2-mMaple | NDC80-GFP::kanMX4, MIF2-mMaple::HIS3MX6 |
| Ndc80-GFP/ Mtw1-mMaple | NDC80-GFP::kanMX4, MTW1-mMaple::HIS3MX6 |
| Spc42-GFP/Ndc80-mMaple | SPC42-GFP::kanMX4, NDC80-mMaple::HIS3MX6 |
| Ndc80-GFP/ Nnf1-mMaple | NDC80-GFP::kanMX4, NNF1-mMaple::HIS3MX6 |
| Ndc80-GFP/ Nsl1-mMaple | NDC80-GFP::kanMX4, NSL1-mMaple::HIS3MX6 |
| Ndc80-GFP/ Spc25-mMaple | NDC80-GFP::kanMX4, SPC25-mMaple::HIS3MX6 |
