# Supplementary material for: Nanoscale structural organization and stoichiometry of the budding yeast kinetochore
Source: J Cell Biol. 2023 Jan 27;222(4):e202209094. doi: 10.1083/jcb.202209094 (PMC9929930; doi:10.1083/jcb.202209094)
Supplement: Table S3 — shows the PCR primers used in this study. [file JCB_202209094_TableS3.docx]

**Table S3. The PCR primers used in this study.**

| **Name** | **Sequence** |
| --- | --- |
| Ask1 S2 | GTTCTGATATTCATCACTAGTAAAAATTGTATGTACTTATTTATTCTAATCGATGAATTCGAGCTCG |
| Ask1 S3 | GAAGTACCAAAGCCTGGGACCATCATTCATTTTTCTACGAATAGACGTACGCTGCAGGTCGAC |
| Ask rev check | GGCATATATCTGGCGCACTT |
| Cnn1 S2 | TGTACATAAATTGCCACTATTTAATTATTTTCTCTA |
| Cnn1 S3 | ACGAATTGCTTCCCTTAGAACTTCAATCAAGAATTGAAAGTTATTTGTTCCGTACGCTGCAGGTCGAC |
| Cnn1 rev check | GGTTAGCCACATCTTTTACACTC |
| Ctf19 S2 | CGGAATCGTTTAAGCAAGCCGTCCAGTTGGCAATGGCAAATGGAACATCAATCGATGAATTCGAGCTCG |
| Ctf19 S3 | GTTAAAGGAGATCTGCAACGTTTGCCTATTCCCGGACATGTACGCCAGGCGTACGCTGCAGGTCGAC |
| Ctf19 rev check | AAGAGAATACTACAGTTTCTCCTTTC |
| Dsn1 S2 | TTACATATGCAGAAGTATCCGATTTTTTTTTGATTTTTTCTTTTACTTCAATCGATGAATTCGAGCTCG |
| Dsn1 S3 | AACAGCTGTTGAAGGGATTAAGTTTATCTTTCAGTAAAAAACTGGATTTACGTACGCTGCAGGTCGAC |
| Dsn1 rev check | CTCCGTCTGGAAAAGTCATATG |
| Mif2 S2 | TATTTCTTCAGTACATAGCATGCATAATGAGAATATTCACATCATAATTAATCGATGAATTCGAGCTCG |
| Mif2 S3 | CTAACGATGACAACGACAAAGAATTAGACAGTACGTTTGACACTTTTGGGCGTACGCTGCAGGTCGAC |
| Mif2 rev check | GTTGTAGGCAAGGATGAAATTAAG |
| Mtw1 S2 | GTGAATACATACATCATATCATAGCACATACTTTTTCCCACTTTATATTAATCGATGAATTCGAGCTCG |
| Mtw1 S3 | TTAGTATAGATATTGAAGAGCCTCAATTGGATTTACTTGATGATGTGTTACGTACGCTGCAGGTCGAC |
| Mtw1 rev check | AGTTCTCGCTCAAACTCTAAG |
| Ndc80 S2 | CTGAGCTTTGCTGTAGATTGCTCGGGTATTATATATCATTTATTTTATTAATCGATGAATTCGAGCTCG |
| Ndc80 S3 | TTGAAGAGTTACGAAATTTGGAGTTTGAAACTGAACATAACGTAACAAATCGTACGCTGCAGGTCGAC |
| Ndc80 rev check | CGTCACGTCCATTTTCTTCC |
| Nnf1 S2 | CATAAAACAGGTTCCTTATACGTCACTTATGAAATTTATACAACTAATCAATCGATGAATTCGAGCTCG |
| Nnf1 S3 | TGCTGGTACAGAGCCTCAATGATATGGTCTTGGAATTGAAGGAAAACTATCGTACGCTGCAGGTCGAC |
| Nnf1 rev check | TTGCGGAAAGTCACGATGTC |
| Nsl1 S2 | TATAGTTATCATTTACCACGAGGTTGACTGTCTTATATGTTATTTATTCAATCGATGAATTCGAGCTCG |
| Nsl1 S3 | GATATAACGTTCAAAAGGTCAAGCGCTTAATGGACTTCCTGGAGGAGGATCGTACGCTGCAGGTCGAC |
| Nsl1 rev check | ACTAAAAGTTATTGGTCTTTCCCC |
| Nuf2 S2 | AAAAAGAAGAAAACACAGAAGGGGGAGTAAAAATAAGTATACCGCTGCTAATCGATGAATTCGAGCTCG |
| Nuf2 S3 | TATCTGGTCATATTAATAAATACATGAATGAAATGCTCGAATATATGCAACGTACGCTGCAGGTCGAC |
| Nuf2 rev check | CTGTTGGGATTCTGTGATGAC |
| Okp1 S2 | TTAGTTATATGCATCGTAATCGTAAACTCTGAAACAATGGATTATCGCTAATCGATGAATTCGAGCTCG |
| Okp1 S3 | TTGTACCGCACCATGAGTCGCACCAAGATAAGACCGAAGAAGATATACACCGTACGCTGCAGGTCGAC |
| Okp1 rev check | CAAAGATTTAATTATTGCTTCCCCTT |
| Spc25 S2 | TCTAAATCATAGGCCCAGAATAAACTGAACAGATGCGTATAAAGGCGTTAATCGATGAATTCGAGCTCG |
| Spc25 S3 | ACCTCGCGGCATTTTTAGTCGTGGCCCGCGATATGCTTCTGGCATCTTTACGTACGCTGCAGGTCGAC |
| Spc25 rev check | GAGAGTGATGTCAATGAGCG |
| Spc105 S2 | TAAAAAAAAGTGATGAGATATTACTAGTCATCGTTGTCCTATTATAAACACTTTAATCGATGAATTCGAGCTCG |
| Spc105 S3 | GATCAGTGGAGTTCTTCCTTCATTTACGAAAAGTAGAATACATTTAGAGTTTACGCGTACGCTGCAGGTCGAC |
| Spc105 rev check | TCGAGCTTTTCTTCAAATTG |
| Cse4 S2 | GGGAAAAATCGGCTCCAGCCCTGAAGGACAAATATCACTAATCGATGAA???GA??TCG |
| Cse4 S3 | AGACATGCAACTAGCAAGAAGAATCAGGGGACAGTTTATTCGTACGCTGCAGGTCGAC |
| Cse4 rev check | CAGAAGTATCCCTTAATCAGTCTG |
| Cep3 S2 | TAACGATTCATCCGCGAGATTTCAAAGGATATGAAATATGGTTGCAGTTAATCGATGAATTCGAGCTCG |
| Cep3 S3 | CTATTGTAGAAGAGTTTCAATCCGAATATAACATTTCTGACATACTTTCCCGTACGCTGCAGGTCGAC |
| Cep3 check rev | TACATCTGATGCATCGTTGTC |
| Chl4 S2 | TTTTTTTTTTTATTGGAAGCGATAACAGAACACAGTAGTTTTCTAATCGATGAATTCGAGCTCG |
| Chl4 S3 | TAATGAATGGAGATTTTCAGCGAGAGCAGGTTGCTAAAGGTGGTTTACTGCGTACGCTGCAGGTCGAC |
| Chl4 rev check | TCCTCCTTATTCACTGGCATG |
| Kan frw check | CAAGACTGTCAAGGAGGG |
| His frw check | CAAGACTGTCAAGGAGGG |
| Hph frw check | AGTTATGTTAGTATTAAGAACG |
