# Supplementary material for: Nanoscale structural organization and stoichiometry of the budding yeast kinetochore
Source: J Cell Biol. 2023 Jan 27;222(4):e202209094. doi: 10.1083/jcb.202209094 (PMC9929930; doi:10.1083/jcb.202209094)
Supplement: Table S4 — shows additional information about the dual-color SMLM experiments. [file JCB_202209094_TableS4.docx]

**Table S4. Additional information about the dual-color SMLM experiments.** For each protein of interest, the number of performed experiments, ROIs and kinetochore spindles are depicted.

| **Protein** | **Number of experiments** | **Number of ROIs** | **Number of kinetochore spindles** |
| --- | --- | --- | --- |
| Ask1 | 12 | 30 | 15 |
| Nuf2 | 5 | 10 | 5 |
| Ndc80 | 18 | 50 | 25 |
| Nsl1 | 7 | 16 | 8 |
| Nnf1 | 6 | 14 | 7 |
| Mtw1 | 15 | 34 | 17 |
| Dsn1 | 12 | 26 | 13 |
| Spc25 | 11 | 26 | 13 |
| Okp1 | 11 | 24 | 12 |
| Ctf19 | 6 | 16 | 8 |
| Cep3 | 6 | 12 | 6 |
| Cse4 | 10 | 20 | 10 |
| Cnn1 | 8 | 16 | 8 |
| Mif2 | 10 | 20 | 10 |
| Ctf19-Ndc80 | 17 | 46 | 23 |
