# Supplementary material for: Nanoscale structural organization and stoichiometry of the budding yeast kinetochore
Source: J Cell Biol. 2023 Jan 27;222(4):e202209094. doi: 10.1083/jcb.202209094 (PMC9929930; doi:10.1083/jcb.202209094)
Supplement: Table S5 — shows calibration factors for protein counting. [file JCB_202209094_TableS5.docx]

**Table S5. Calibration factors for protein counting.** The factor is the ratio between number of localizations and the copy number of Nup188 (16 copies) per NPC.

| **Protein** | **Replicate** | **Calibration factor** |
| --- | --- | --- |
| Ask1 | 1 | 0.89 |
|  | 2 | 1.02 |
| Ndc80 | 1 | 1.22 |
|  | 2 | 1.56 |
| Spc105 | 1 | 1.26 |
|  | 2 | 1.29 |
| Dsn1 | 1 | 1.15 |
|  | 2 | 1.51 |
| Chl4 | 1 | 1.42 |
|  | 2 | 1.17 |
| Ctf19 | 1 | 1.20 |
|  | 2 | 1.50 |
| Cnn1 | 1 | 1.14 |
|  | 2 | 1.34 |
| Mif2 | 1 | 1.15 |
|  | 2 | 1.25 |
| Cep3 | 1 | 1.46 |
|  | 2 | 1.22 |
| Cse4-i | 1 | 1.38 |
|  | 2 | 1.24 |
| Cse4 | 1 | 1.20 |
|  | 2 | 1.23 |
